# A case report of allergic blepharoconjunctivis to alcaftadine

**DOI:** 10.1186/1939-4551-8-S1-A279

**Published:** 2015-04-08

**Authors:** Leda Das Neves Almeida Sandrin

**Affiliations:** 1 Sociedade Brasileira De Alergia e Imunopatologia,Brazil; 2 Unochapecó, Brazil; 3 Faculdade De Medicina Da Universidade De São Paulo, Brazil

## Background

Alcaftadine is a novel drug available in eye drops to treat allergic conjunctivitis.

## Methods

This is a case report.

## Results

A 32-year-old Brazilian woman presented with chronic pruritic eyes for three years and recurrent episodes of eyelid inflammation during the last six months in an ophthalmology practice. The clinical finds were conjunctival hyperemia and edema of the eyelids. The hypothesis of perennial allergic conjunctivitis was done and the use of alcaftadine eye drops once a day was prescribed. She returned after two weeks and the worsening of the symptoms was related. She was advised to visit an allergist and to stop the use of alcaftadine. At that time she had edema and erythema of both eyelids. Allergic contact blepharoconjunctivitis was suspected and standardized patch test as well as products she was using on the face, on the hair and over the eyes were tested. Alcaftadine (+++48h/+++96h), hidroquinone (+++48h/+++96h), fragrance mix (++48h/+96h), triethanolamine (++48h/+96h), shampoo (+++48h/+++96h) were positive. Benzalkonium chloride, a preservative found in alcaftadine, was negative. SPT to grass pollen and house dust mites were positive. Avoidance of alcaftadine and shampoo, use of preservative free eye lubricants, nasal corticosteroids and environmental control had completely cleared the symptoms.

## Conclusions

Ocular allergies encompass a number of inflammatory diseases in the ocular surface which have different hypersensitivity mechanisms and occur in 20% of population. The different syndromes can coexist and it is very important to be aware about the possibility of drug sensitization in these cases. Furthermore, a multidisciplinary approach by allergists and ophthalmologists is required to fully understand these patients.

## Consent

Written informed consent was obtained from the patient for publication of this abstract and any accompanying images. A copy of the written consent is available for review by the Editor of this journal.

